# Lighting and circadian cues shape locomotor strategies for balance and navigation in larval zebrafish

**DOI:** 10.1098/rspb.2026.1651

**Published:** 2026-10-14

**Authors:** Jiahuan Liu, Andy Kraja, Samantha N. Davis, Hannah Gelnaw, Naoroz Mahmood, David Schoppik, Yunlu Zhu

**Affiliations:** ^1^ Departments of Neuroscience, Otolaryngology, and the Institute for Translational Neuroscience, NYU Grossman School of Medicine, New York, NY, USA; ^2^ Department of Psychology, New York University, New York, NY, USA; ^3^ Courant Institute of Mathematical Sciences, New York University, New York, NY, USA; ^4^ Center for Neural Science, New York University, New York, NY, USA; ^5^ Kresge Hearing Research Institute, Department of Otolaryngology-Head and Neck Surgery, University of Michigan, Ann Arbor, MI, USA; ^6^ Department of Molecular and Integrative Physiology, University of Michigan, Ann Arbor, MI, USA

**Keywords:** locomotion, postural control, zebrafish, motor behaviour, vestibular system, circadian, biological clock

## Abstract

Most fish are inherently unstable and must swim to stabilize posture. How diurnal fish reduce activity at night while maintaining postural control remains unclear. We defined distinct locomotor strategies that larval zebrafish (*Danio rerio*) use to control posture and navigate the water column in response to light and circadian cues. In the dark, larvae maintain balance by swimming in long bouts with large nose-up rotations, compensating for nose-down drift accrued during prolonged inactivity. Effective postural compensation requires vestibular sensation from the utricle. By contrast, in the light, larvae navigate with short, frequent and variable bouts. While lighting exerts a dominant, masking effect on the locomotor strategies, circadian rhythms modulate swim kinematics. Our results reveal distinct light–dark locomotor strategies and disentangle how ambient light and the internal clock jointly shape balance control and navigation. This work lays the foundation for understanding how external and internal cues interact to govern locomotor activity in freely moving diurnal animals.

## 1. Introduction

Environmental lighting and the internal circadian clock interact to regulate most animals' locomotor activity. The internal clock drives rhythmic behaviour [1], while light acts both as a dominant zeitgeber that entrains the clock [2] and as a direct modulator of behaviour through its masking effects [3–5]. Terrestrial animals that are active during the day rest at night by remaining still [6,7]. However, zebrafish (*Danio rerio*), like most fish species, lack a stable body plane [8,9], requiring active swimming to maintain their preferred horizontal posture [10]. How diurnal fish stabilize posture despite reduced locomotor activity at night remains unknown.

Larval zebrafish are an ideal system for studying how lighting and circadian rhythms shape locomotor behaviour. They are more active during the day [11–17] and display light-induced masking of activity [18,19]. Zebrafish larvae swim in discrete bouts separated by periods of inactivity [20,21], making their locomotion easy to quantify. Owing to their front-heavy body plane [9,10], larvae must correct body tilts using vestibular feedback [10,21,22] to maintain preferred horizontal posture and navigate the water column effectively. Although lighting and circadian cues influence activity levels in zebrafish larvae, their specific impact on the kinematics governing postural control and navigation remains unexplored.

We combined high-resolution behavioural recordings with photoperiodic manipulations to dissect how lighting and circadian rhythms shape locomotor strategies in larval zebrafish. We found that larvae adopt two distinct strategies depending on light availability and identified bout duration as a key kinematic parameter that classifies these two strategies. In the dark, they prioritized postural stabilization though pronounced nose-up rotations that compensated for nose-down postural drifts accrued during prolonged inactivity. In the light, larvae adopted frequent, short swim bouts with high directional variability, facilitating horizontal exploration. Interestingly, while lighting exerts a dominant masking effect on locomotor strategies, circadian rhythms modulate swim kinematics. Postural control and navigation exhibited rhythmic fluctuations in constant darkness. In conclusion, our study disentangles the contributions of lighting and circadian rhythms to locomotor activity, revealing how a simple vertebrate adjusts locomotor strategies to balance and navigate in response to external and internal cues.

## 2. Material and methods

### (a) Fish husbandry

Adult zebrafish were maintained at 28.5${}^{\circ }$C under a standard 14/10-hour light/dark (LD) cycle with lights on from 09.00 to 23.00. Fertilized embryos were derived from in-crosses of wild-type zebrafish with a mix of AB/WIK/TU/SAT/NHGRI-1 backgrounds. Larvae were kept in incubators set to a 28.5${}^{\circ }$C 14/10-hour LD cycle until behavioural assessment.

### (b) Behavioural measurements

We used the scalable apparatus to measure posture and locomotion (SAMPL) to record behaviour of freely swimming zebrafish larvae [20]. The apparatus, experimental procedure, program and analysis pipeline have been detailed previously [20]. Each behaviour box was equipped with a cool white LED strip (Amazon B075R4X1XL) to control photoperiodic treatments, illuminating the chamber with cool white light measured approximately between 50 to 150 lux with irradiance between 0.61 to 3.91 W m^–2^. Constant light (LL) and constant dark (DD) conditions were generated by keeping the white LED on or off, respectively. For the LD condition, the white LED followed a standard 14/10-hour LD cycle with the light on from 09:00 to 23:00.

For 2-day recordings, five to seven larvae at 7 days post-fertilization (dpf) were transferred into each behaviour chamber containing 30 ml of E3 medium, filling an approximately 50 x 50 x 13 mm^3^ water volume. Each chamber was positioned between a camera and a 940 nm infrared light source inside a light-tight box. Coordinates and pitch-axis body angle were recorded at 166 Hz for approximately 48 hours, starting before 11.00 in a field of view of 20 x 20 mm^2^. Larvae were fed cultured rotifers (Reed Mariculture) after approximately 24 hours. Data were collected from 105 and 98 fish under dark and light conditions, respectively, across five experimental repeats, with three SAMPL boxes containing approximately 20 fish per condition per repeat. Each repeat contained larvae of the same clutch (siblings). Additional sample-size information is provided in the figure legends and tables.

For the 6-day recordings used for circadian analyses, each chamber contained only a single larva. Recordings began at 4 dpf. Each chamber was supplemented with 3 ml of rotifer culture and covered with a piece of Parafilm punctured with holes to reduce evaporation. Recordings were performed for 6 days without additional feeding or opening of the boxes. This dataset was collected from 18, 19 and 17 fish under DD, LD and LL conditions, respectively. Additional information on sample size is provided in the figure legends and tables.

### (c) Lesions of lateral-line hair cells

At 7 dpf, larvae were treated with 10 µM copper sulfate (CuSO4) in E3 medium for 90 minutes. Control siblings were handled identically and transferred to E3. After the treatment, larvae were washed in E3 and transferred to behavioural chambers. The CuSO_^4^_ treatment was repeated after 24 hours of behavioural recording to prevent hair cell regeneration. Quantification of hair cell loss following the treatment can be found in [23].

### (d) Behavioural analysis

Raw behavioural data were preprocessed using the previously published SAMPL analysis pipeline [20]. Time-series data were segmented into swim bouts (peak speed > 5 mm s^–1^) and inter-bout intervals (IBIs). Behavioural parameters were defined as follows: bout duration, time during which swim speed remained above 50% of peak speed; swim displacement, Euclidean distance travelled while swim speed exceeded 5 mm s^–1^; swim direction, instantaneous trajectory on the pitch axis at peak speed; bout rotation, net change in pitch angle from –250 ms to +200 ms relative to peak speed; IBI postural drift, pitch change between the end of one bout and the start of the next; postural compensation residual, sum of IBI postural drift and the subsequent bout rotation; residual variability, median absolute deviation of the compensation residual. Navigation metrics were calculated from epochs spanning five consecutive swim bouts: displacement per second, Euclidean distance between the starting positions of the first and fifth bouts divided by the elapsed time; directional change, absolute change in swim direction between consecutive bouts [21]; directional change per second, cumulative absolute directional changes between bouts 1 and 5 divided by the elapsed time.

To visualize circadian effects, we extracted data from 09.00 on the second day of behaviour (5 dpf) to 09.00 on the sixth day (9 dpf). Kinematic measurements were binned into 1-hour intervals and summarized as the median value for each fish. The binned values were pooled and detrended by subtracting the fitted linear drift estimated by linear regression. Lomb–Scargle periodograms were then applied to the detrended values, with the peak period searched over 12 to 36 hours. The dominant period identified for each condition was used for subsequent Cosinor fitting.

### (e) Statistical analysis

All statistical analyses were performed using the SciPy package in Python [24]. Code and usage instructions are available in the data repository [25]. Swim parameter distributions were assessed for normality and none were normally distributed. Thus, non-parametric methods were used for analyses performed at the swim-bout level. Statistical comparisons at the level of repeats were performed on repeat medians using independent *t*-tests. An experimental repeat consisted of sibling larvae from a single cross recorded simultaneously under all conditions. For figures 1–3, swim bouts were pooled within each repeat, summarized by the median and compared across conditions using independent *t*-tests on repeat medians. Complementary tables report pooled-bout distributions as medians with interquartile ranges (IQR). *P*-values were calculated using the median test, effect sizes were estimated as $\sqrt{\chi^{2}/N}$ [26] and Scott's rule determined histogram bin numbers [27].

**Figure 1.**
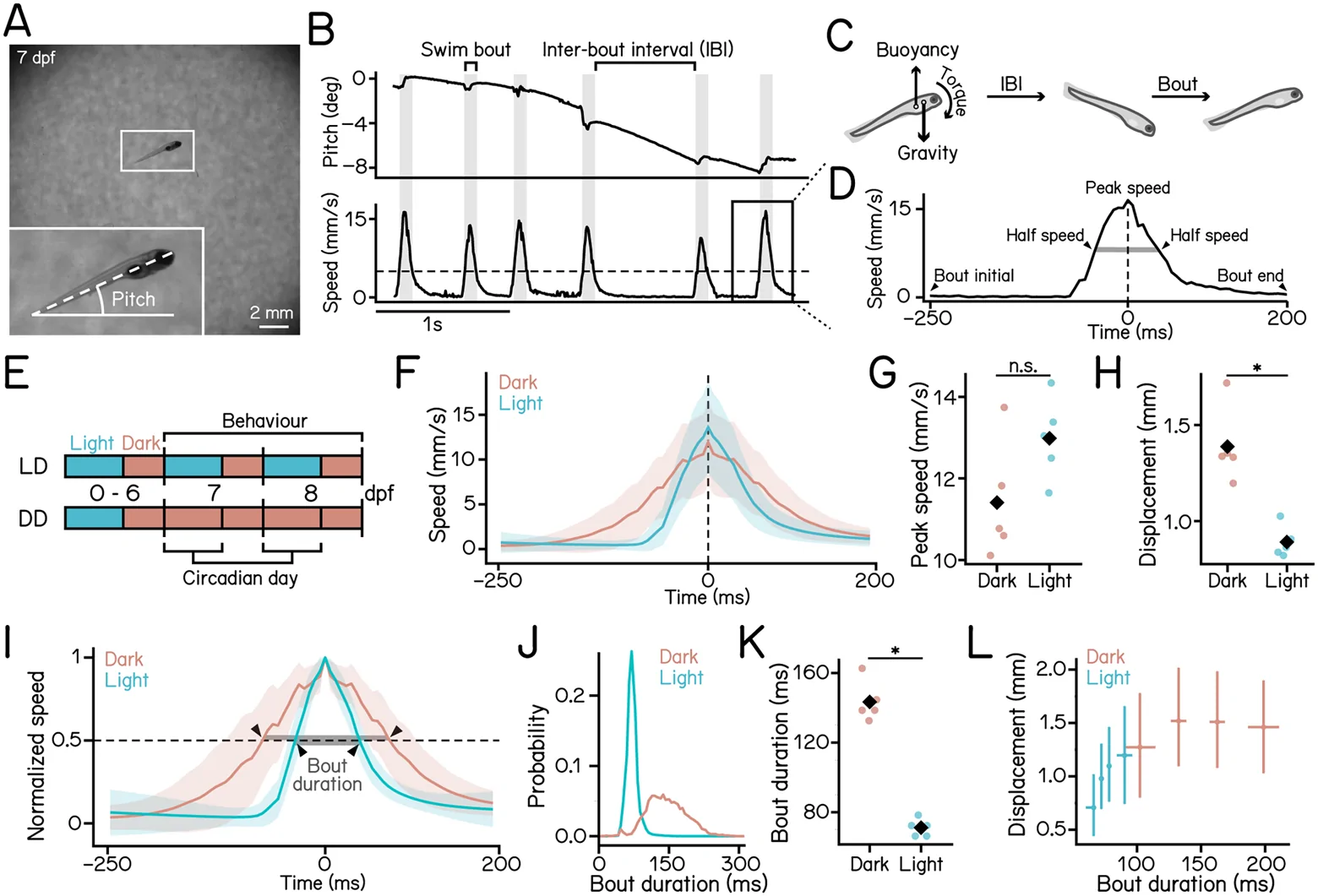
Measuring posture and locomotion in freely swimming zebrafish larvae reveals distinct kinematics in dark and light. (A) Representative 7 dpf larva recorded in SAMPL. Pitch angle is the trunk angle relative to horizontal; positive values indicate nose-up posture. (B) Example epoch showing pitch angle and swim speed; dashed line marks the 5 mm s^–1^ bout-detection threshold; grey bars indicate bouts. (C) Schematic of vertical forces. An anterior centre of mass relative to centre of buoyancy causes nose-down rotation during IBIs, corrected by swim bouts. (D) Swim-speed time series for one bout. Dashed line marks peak speed; bout duration is the width at half maximum (grey bar, arrowheads). (E) Experimental paradigm. Larvae were raised under a 14 : 10 LD cycle and recorded from 7–9 dpf under LD or DD conditions. Photophase LD and subjective-day DD data were analysed. (F) Swim speed plotted over time in dark (red) and light (cyan) conditions. Lines show means across 5 repeats; shading indicates ±  1 SD. (G and H) Peak swim speed (G) and bout displacement (H) in dark and light. Dots show repeat medians; black diamonds show group means. (G) *p* = 8.053e-02, Cohen's *d* = 1.265; (H) *p* = 7.831e-04, Cohen's *d* = 3.314; *t*-tests. (I) Normalized swim-speed time series. Lines show means across five repeats; shading indicates ±  1 SD. Bout duration is the width at half maximum. (J and K) bout-duration histogram (J) and repeat-level quantification (K) in dark and light. Dots show repeat medians; black diamonds show group means. *p* = 1.315e-06, Cohen's *d* = 8.090; *t*-test. (L) Bout displacement versus bout duration. Data were grouped into bout duration quartiles; crosses show medians and error bars show IQRs. *n* = 23 875/98 722 bouts from 105/98 fish for dark/light over five experimental repeats. See also in table S1 in the supplementary material.

The relationship between bout rotation and IBI drift was quantified using bi-square regression. Correlation coefficients were compared using two-way ANOVA followed by Tukey HSD tests with Bonferroni correction.

For the 6-day circadian recordings, each chamber contained a single larva; therefore, all analyses were performed at the individual-fish level. Lomb–Scargle periodogram significance was assessed by permutation testing where hourly median values for each fish were randomly reassigned to their original time labels 1000 times to generate a null distribution of maximum periodogram power, and *p*-values were calculated as the proportion of permutations exceeding the observed maximum power.

## 3. Results

### (a) Larvae employ distinct swimming kinematics under dark and light

We first examined how lighting affects kinematics of freely swimming zebrafish larvae. We recorded larval activity from their side (figure 1A) using a custom-designed SAMPL [20]. Zebrafish larvae swim in short, discrete bouts interspersed with inactive periods called IBIs (figure 1B). Owing to their anteriorly positioned centre of gravity relative to the centre of buoyancy (figure 1C), larvae are inherently unstable and rotate nose-down during IBIs [10] (figure 1B,C). We recorded swim parameters from 7 to 9 days post-fertilization (dpf) and extracted a 450 ms window of activity for each bout (figure 1D). We generated LD and dark–dark (DD) photoperiodic conditions by turning the daylight LED in the apparatus on or off during the subjective day, respectively (figure 1E).

Compared to siblings under light conditions, larvae in the dark reached a comparable peak speed (figure 1F,G; dark: 11.409 ±  1.445 mm s^–1^ versus light: 12.982 ± 1.003 mm s^–1^, *p* = 8.053e-02) and exhibited greater swim displacement (figure 1H; dark: 1.388 ± 0.195 mm versus light: 0.891 ±  0.082 mm, *p* = 7.831e-04). The difference in displacement was significant, whereas peak speed did not differ significantly, probably reflecting limited statistical power from the small number of experimental repeats. To complement the repeat-level analysis, we compared bout-level distributions, which showed lower peak speeds but greater displacement in the dark (supplementary material, table S1).

Notably, we found that dark bouts exhibited more gradual changes in speed (figure 1F). To quantify the dynamics of the speed profile, we normalized each swim bout by its peak speed and measured the bout duration, defined as the duration during which larvae moved at speeds greater than half their peak speed (figure 1I). Larvae in dark showed significantly longer bout durations compared with their siblings in light (figure 1J,K ; dark: 143.373 ± 11.588 ms versus light: 71.084 ± 5.040 ms, *p* = 1.315e-06).

These findings led us to hypothesize that larvae achieve greater displacement through longer bouts. We plotted displacement as a function of bout duration and observed a positive correlation in the light condition (figure 1L, cyan), indicating that increased bout duration is associated with greater displacement. However, this relationship was absent in bouts recorded in the dark (figure 1L, red), suggesting that larvae employ a different locomotor regime without light, and leaving open the question of the functional significance of extending bout duration in the dark.

These results demonstrate that zebrafish larvae exhibit distinct swim kinematics in light versus dark conditions, and that *bout duration* reliably differentiates activity between these conditions.

### (b) Swim bouts in the dark stabilize posture

Zebrafish larvae rotate in the pitch axis during swim bouts [10]. Longer bout durations allow more time for rotation. Given the significantly longer bout durations in the dark, we hypothesized that larvae use these extended swim bouts to achieve greater body rotation.

We measured rotations during swim bouts (figure 2A) and observed significantly greater nose-up rotations in dark bouts (figure 2B,C; dark: 5.724 ± 3.042${}^{\circ }$, light: 1.053 ± 0.504${}^{\circ }$, *p* = 9.538e-03). Notably, bout rotations were positively correlated with bout duration in the dark (figure 2D, red), indicating that larvae extend their bout duration to achieve greater nose-up rotations.

**Figure 2.**
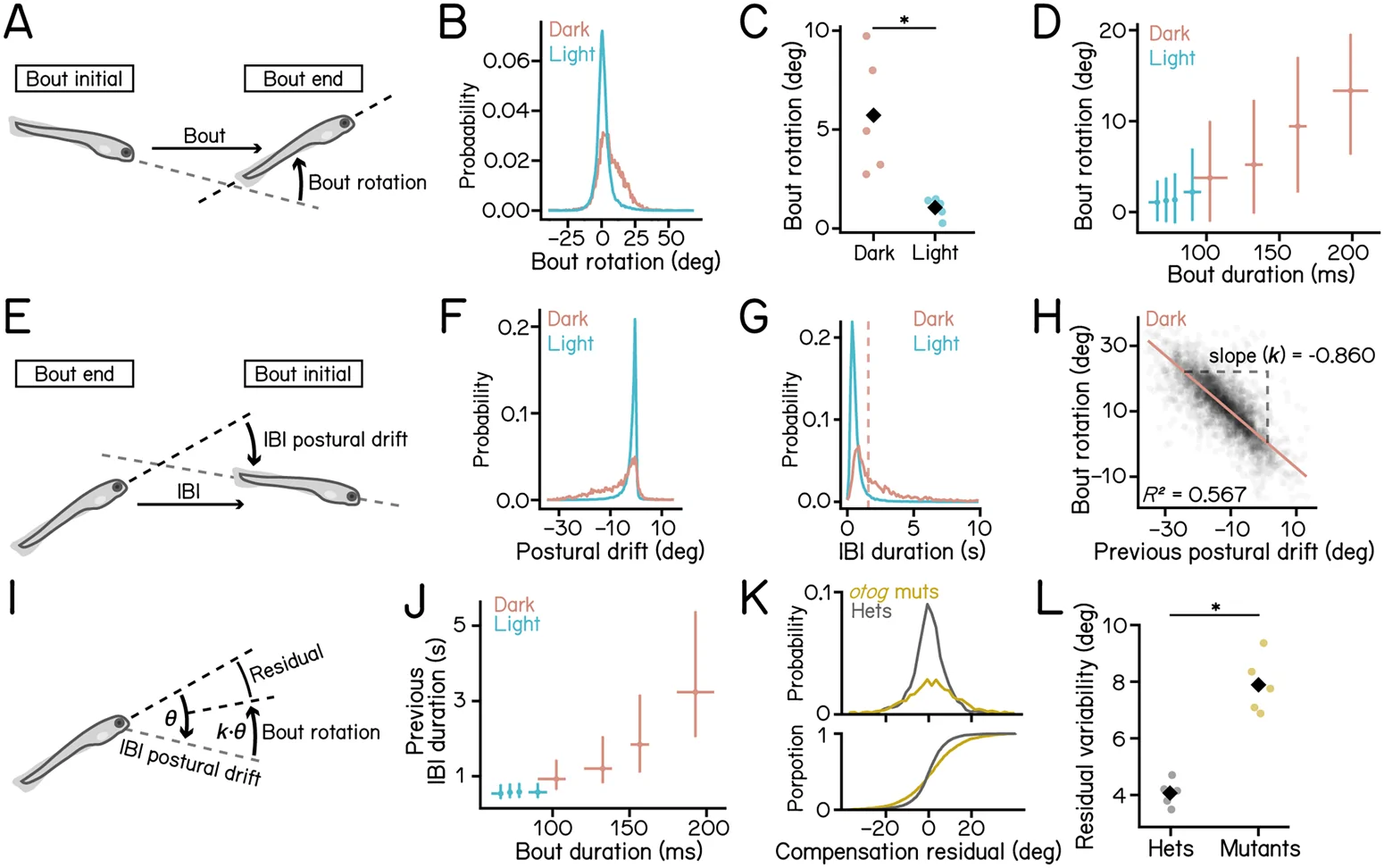
Swim strategy in dark compensates for postural drifts accrued during prolonged inactivity.(A) Schematic illustration of bout rotations, defined as the change in pitch angle during a swim bout. (B) Histogram of bout rotation under dark (red) and light (cyan) conditions. (C) Bout rotation under dark (red) and light (cyan) conditions. Dots show repeat medians; black diamonds show group means. *p* = 9.538e-03, Cohen's d = 2.142, *t*-test. (D) Bout rotation versus bout duration. Data were grouped into bout duration quartiles; crosses show medians and error bars show IQRs. (E) Schematic illustration of IBI postural drift, defined as the change in the pitch angle from the end of the previous bout to the beginning of the current bout. (F) Histogram of postural drift under dark (red) and light (cyan) conditions. (G) Histogram of postural drift under dark (red) and light (cyan) conditions. Dashed red line marks the median dark IBI duration. Bouts with longer than this median are classified as long IBIs. (H) Bout rotation after long IBIs versus preceding IBI postural drift in the dark. Red line shows robust bi-square regression fit: slope *k* = −0.860, $R^{2}$ = 0.567. (I) Schematic illustration of compensation for postural drift following long IBIs. Larvae rotate nose-up proportionally to the amount of nose-down drift during the previous IBI. (J) Previous IBI duration versus bout duration. Data were grouped into bout-duration quartiles; crosses show medians and error bars show IQRs. (K) Histogram (upper) and cumulative distribution (lower) of compensation residuals of *otog*^–/–^ mutants (muts; yellow) and heterozygous control (Hets; grey). Bouts in the dark with long proceeding IBIs were plotted. (L) Residual variability in *otog*^–/–^ mutants and controls. Dots show repeat medians; black diamonds show group means. *p* = 5.652e-05, Cohen's *d* = 4.881; *t*-test. *n* = 23 875/98 722 bouts from 105/98 fish for dark/light over five repeats. *n* = 3143/34 285 middle bouts from three-bout sequences were selected for calculation of IBI postural drifts for dark/light conditions. For the *otog* mutant dataset, *n* = 3106/1716 bouts with long preceding IBIs from 99/136 fish for Hets/mutant over five repeats. See also in figure S1 and tables S1and S2 in the supplementary material.

Why do they rotate more nose-up in the dark? Because larvae experience a constant nose-down torque, we reasoned that, in the dark, they would accumulate larger nose-down postural drifts during inter-bout intervals (figure 1C). We quantified kinematics during IBIs (figure 2E) and found that larvae in the dark showed increased nose-down drifts (figure 2F) and prolonged IBI durations (figure 2G). These results support the hypothesis that larvae rotate nose-up to compensate for nose-down drifts accrued during prolonged IBIs in the dark.

To directly test this hypothesis, we examined correlations between IBI postural drift and subsequent bout rotation. We categorized swim bouts into those with long versus short IBIs based on the median dark IBI duration (figure 2G, dashed vertical line). We plotted bout rotations against IBI postural drifts during either the preceding or following IBI. Bout rotations in the dark were negatively correlated with postural drift during long IBIs, whereas no such correlation was observed under the light condition (supplementary material, figure S1A–C). Interestingly, bout rotations were strongly linearly correlated with drift during the preceding IBIs (figure 2H, $R^{2}$ = 0.567), but not the following IBIs (supplementary material, figure S1B,D, $R^{2}$ = 0.172), suggesting that larvae compensate for prior postural drift rather than anticipate future drift. To measure the degree of compensation, we defined the absolute slope of the best-fit line as the compensation gain (figure 2H). Swim bouts in the dark exhibited a gain of 0.860 (figure 2H), indicating that following long IBIs larvae effectively compensate for nose-down rotations accrued during the IBI through swim bouts (figure 2I). Correspondingly, bouts after longer IBIs in the dark exhibited longer bout durations (figure 2J). These results demonstrate that larvae perform nose-up counter rotations to compensate for nose-down drifts accrued during long periods of inactivity.

Building upon this observation, we further examined whether the IBI duration or the magnitude of postural drift determines the amount of compensatory rotation. Fish rely on their lateral line to sense water flow and the water–air interface [28,29]. Lateral line sensation is critical for many locomotor behaviours including predator detection, prey capture, schooling and rheotaxis [30–33]. Recent work showed that ablation of lateral-line hair cells after swim bladder inflation in larval zebrafish reduces the size of the swim bladder and makes them more negatively buoyant [23]. To dissociate IBI duration from the amount of passive postural drift, we took advantage of this balance challenge that exacerbates the nose-down destabilizing torque. Low-concentration copper sulfate ablation of lateral-line hair cells increased nose-down angular velocity during inactivity (supplementary material, figure S1E). Although lesioned larvae exhibited shorter IBIs, they accumulated greater nose-down drift and generated larger nose-up bout rotations (supplementary material, figure S1F– H). Excitingly, we observed that bout rotation remained linearly related to preceding IBI drift (supplementary material, figure S1I and table S2, $R^{2}$ = 0.545) with a compensation gain of 0.756. The robust compensation for postural drift following lateral-line hair cell ablation suggests that this behaviour remains largely intact despite the loss of lateral-line input. We conclude that compensatory rotation scales with the amount of passive postural drift accrued, rather than with the duration of the inactive period.

Next, we asked whether the sensation of gravity contributes to the compensatory rotation. We used the *otogelin* mutant [34], which lacks the utricular otolith for the first two weeks of life (supplementary material, figure S1J) and thus exhibits impaired gravity sensation [35,36]. We selected mutant larvae with successfully inflated swim bladder. We found no significant differences in basic locomotor parameters between mutants and heterozygous siblings. Mutant larvae exhibited comparable swim speed (12.813 ± 0.782 mm s^–1^ versus 12.847 ± 1.217 mm s^–1^, *p* = 9.601e-01), bout duration (107.229 ± 11.588 ms versus 103.614 ± 20.606 ms, *p* = 7.412e-01), IBI duration (1.589 ± 0.534 s versus 1.243 ± 0.671 s, *p* = 3.942e-01) and IBI postural drift (−2.146 ± 1.787 deg versus −1.033 ± 1.102 deg, *p* = 2.700e-01). To assess the effectiveness of compensatory rotation, we examined their bouts following long IBIs and quantified the compensation residual, defined as the sum of IBI postural drift and the bout rotation (figure 2I). Mutants showed a broader distribution of compensation residual than their heterozygous siblings (figure 2K), reflected in significantly higher residual variability (figure 2L; sibs: 4.070 ± 0.458${}^{\circ }$, mutants: 7.892 ± 1.008${}^{\circ }$, *p* = 5.652e-05). These results show that vestibular sensation via the inner-ear otolith is essential for effective postural compensation.

We conclude that larvae in the dark use long swim bouts with pronounced nose-up rotations to compensate for nose-down postural drifts during prolonged inactivity, reflecting a locomotor strategy to stabilize posture.

### (c) Locomotor strategy in the light favours exploration

Previous studies reported that light increases the activity level and swim distance of larval zebrafish [15], thereby promoting effective exploration [37]. By contrast, we found that although larvae initiated swim bouts more frequently in the light (figure 2G), individual bouts generated shorter displacements than those in the dark (figure 2D). This discrepancy prompted us to examine how larvae chain swim bouts to explore the water column in the light.

We compared swim trajectories between light and dark conditions and observed that there were more swim bouts and longer distances were travelled overall in the light (figure 3A). We quantified swim displacement efficiency as displacement per second (§2d). Larvae in the light travelled significantly further per unit time (figure 3B,C; dark: 2.317 ± 0.831 mm s^–1^, light: 3.980 ± 0.556 mm s^–1^, *p* = 5.882e-03, see also supplementary material, table S3). Larvae primarily swim horizontally in the light while performing more climbs in the dark (figure 3D). Thus, larvae travel further in the light by combining short bouts with higher bout rates.

**Figure 3.**
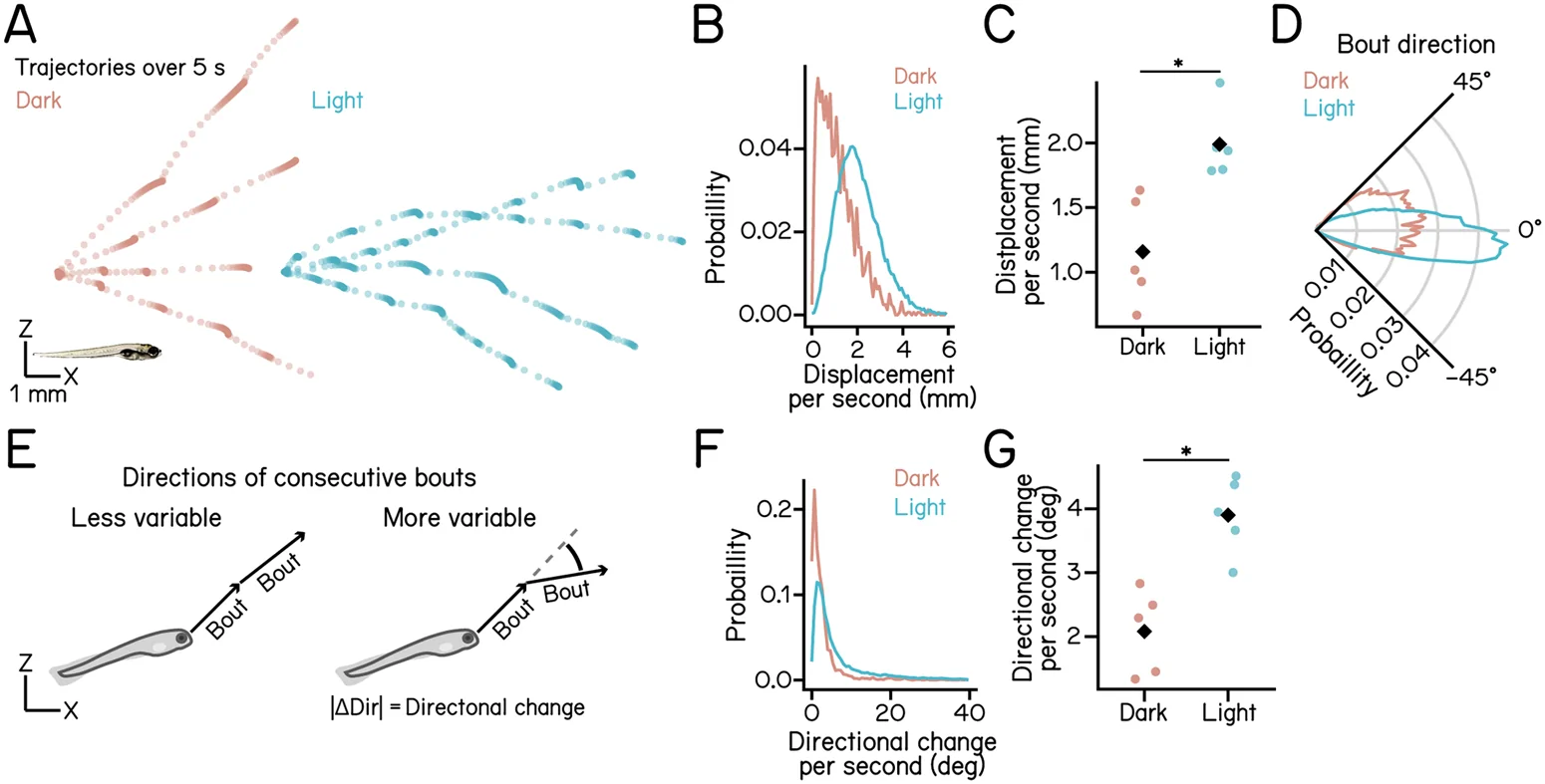
Swim bouts in the light travel further with greater directional variability per unit time. (A) 5 s larval trajectories in dark (red) and light (cyan). Four left-to-right trajectories are shown per condition; dots mark positions every 30 ms. Larva shown for scale. (B) Histogram of Euclidean displacement per second under dark (red) and light (cyan) conditions. (C) Per second Euclidean displacement under dark (red) and light (cyan) conditions. Dots show repeat medians; black diamonds show group means. *p* = 5.882e-03, Cohen's *d* = 2.352; *t*-test. (D) Polar histogram showing distributions of bout directions. (E) Schematic of directional change across bouts, defined as the summed absolute change in swim direction between consecutive bouts. (F) Histogram of directional change per second under dark (red) and light (cyan) conditions. (G) Per second directional change under dark (red) and light (cyan) conditions. Dots show repeat medians; black diamonds show group means. *p* = 1.862e-03, Cohen's *d* = 2.881; *t*-test. *n* = 7980/120 010 sets of consecutive bouts from 105/98 fish for dark/light over five experimental repeats. *n* = 23 875/98 722 swim bouts for (D). See also in table S3 in the supplementary material.

The ability to change movement direction is essential for environmental exploration. Previously, we measured changes in swim directions on the vertical axis across consecutive bouts (figure 3E) and found that larvae in the dark swim with stable trajectories, enabling effective climbs and dives [21]. Here, we examined how short, frequent swim bouts in the light influence changes of swim directions.

We first calculated the average change in direction between adjacent bouts. Bouts in the light showed slightly smaller directional changes than those in the dark (dark: 5.541 ± 0.701${}^{\circ }$, light: 4.123 ± 0.668${}^{\circ }$, *p* = 1.129e-02). We then assessed how directional changes accumulate over time. Interestingly, larvae in the light exhibited significantly greater directional changes per unit time than those in the dark (figure 3F,G; dark: 2.080 ± 1.315${}^{\circ }$ s^–1^, light: 3.901 ± 1.211${}^{\circ }$ s^–1^, *p* = 1.862e-03). This reflects the shorter bout intervals in the light (figure 2G), which increase bout frequency and thus cumulative directional change per unit time despite smaller changes per bout.

Together, these results indicate that, in the light, larvae use short, frequent bouts to achieve greater displacement and directional variability per unit time, a strategy that favours exploration.

### (d) Dissociation of circadian and lighting effects on locomotion

The circadian clock internally regulates locomotor activity. We hypothesized that kinematics of postural control and navigation are rhythmically modulated by the circadian clock.

To capture circadian effects, we adapted a previously established paradigm [14], where embryos and larvae were exposed to LD cycles for 4 days and then transferred to the behavioural apparatus under LD, DD and LL conditions (figure 4A). We then examined their behaviour over 5 days from 5 to 9 dpf.

**Figure 4.**
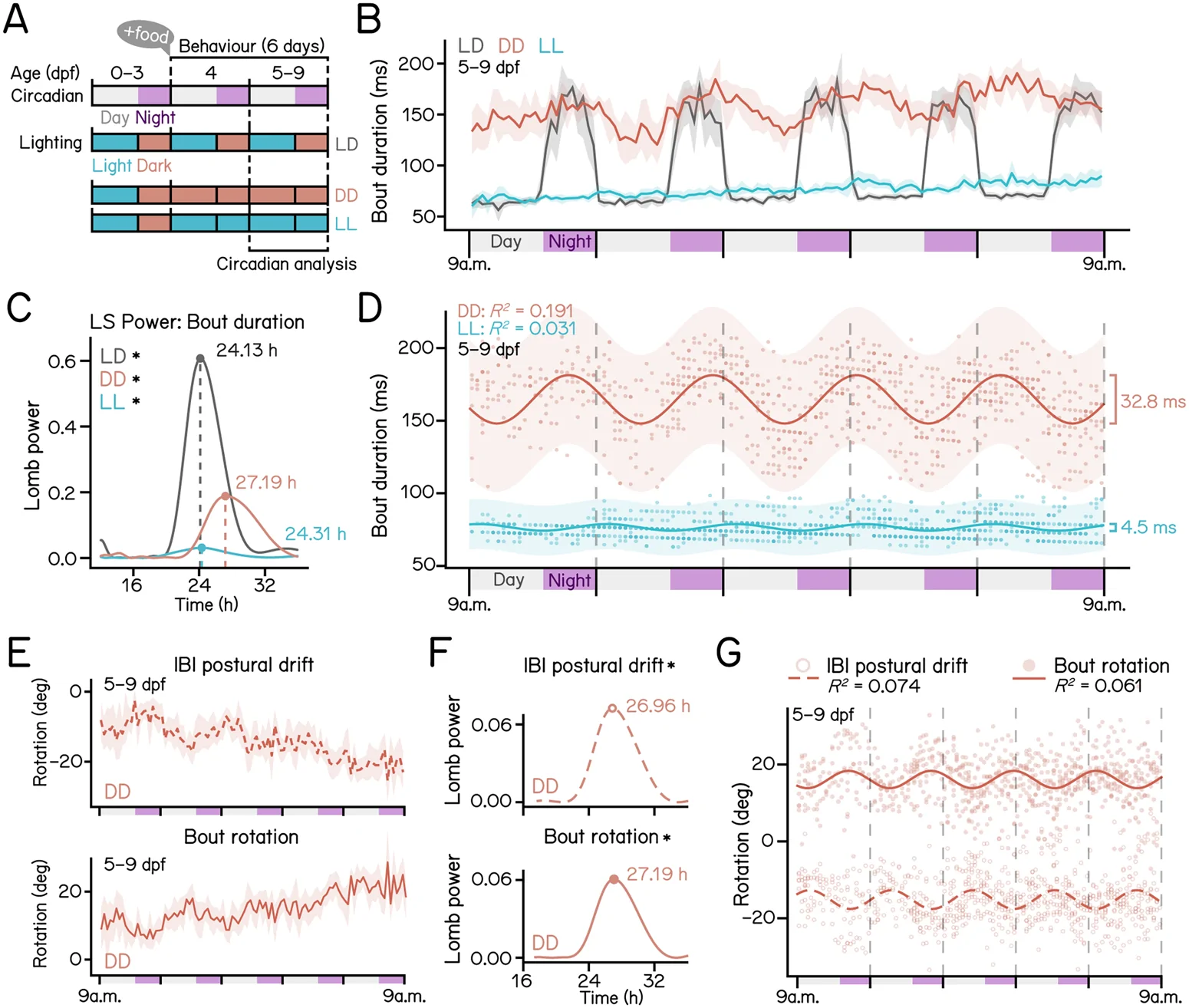
Dissociation of lighting and circadian effects on swim kinematics. (A) Experimental paradigm for circadian analysis. Data from 5 to 9 dpf were analysed. (B) Bout duration over time under DD (red), LD (grey) and LL (cyan). Values are 1-hour binned medians; lines show means and shading indicates 95% confidence intervals. (C) Lomb–Scargle periodograms of detrended bout duration under DD, LD and LL. Dominant peak significance was assessed by permutation tests; *p* < 0.001 for all conditions. (D) Cosinor fits of detrended bout-duration time series under DD (red) and LL (cyan). Bout durations were binned into 1-hour intervals and summarized as per-fish medians. Single-component models were fit at the condition level with the period fixed to the dominant Lomb–Scargle period. Shading indicates 95% confidence intervals. (E) IBI postural drift and bout rotation over time in DD. Values are 1-hour binned medians; lines show means and shading indicates 95% confidence intervals. (F) Lomb–Scargle periodograms of detrended IBI postural drift and bout rotation in DD; permutation *p* < 0.001 for both. (G) Cosinor fits of detrended bout-rotation time series under DD. *n* = 40 080/135 975/115 146 DD/LD/LL bouts over 18/19/17 larvae. For calculation of IBI postural drift in panel (E–G), *n* = 23 791 bouts with a recorded preceding bout over 18 larvae. See also in table S4 in the supplementary material.

Larvae in constant darkness exhibited oscillations in bout duration (figure 4B, red). Strikingly, larvae under LD exhibited a pronounced biphasic pattern with rapid transitions at LD switches (figure 4B, black). By contrast, larvae in LL maintained short bout durations (figure 4B, cyan), indicating a masking effect of light.

To characterize circadian periods, we detrended bout duration measurements to mitigate developmental effects and applied Lomb–Scargle periodogram analysis (figure 4C). We observed strong dominant periods of 27.19 and 24.13 hours under DD and LD conditions, respectively, with powers of 0.19 and 0.61 (permutation *p* < 0.001). The >24 hours period in DD probably represents the free-running period of larval zebrafish. Interestingly, the detected period was approximately 1 hour longer than previously reported [11,14]. By contrast to the robust rhythmic changes in bout duration observed under DD and LD, larvae under LL exhibited only a weak rhythmicity (period = 24.31 hours, power = 0.03, permutation *p* < 0.001).

We further characterized circadian rhythmicity by applying Cosinor analysis using the dominant Lomb–Scargle period. DD larvae exhibited clear oscillations in bout duration, with longer bout duration during circadian night (figure 4D, red; *R*^2^ = 0.191, amplitude = 16.4 ms). By contrast, LL larvae showed poor Cosinor fits (figure 4D, cyan; *R*^2^ = 0.031, amplitude = 2.3 ms). This difference in bout duration, which is merely 4.5 ms between circadian day and night, was smaller than the 6 ms temporal resolution limit of SAMPL [20].

Because longer bouts are associated with greater bout rotations that can compensate for larger postural drift (figure 2), we next asked whether circadian oscillations in bout duration reflected corresponding rhythms in IBI postural drift and bout rotation. Across 5 days of behavioural recordings in DD larvae, IBI postural drift gradually decreased, whereas bout rotation gradually increased (figure 4E). To isolate rhythmic components independent of long-term trends, we detrended these measurements and applied Lomb–Scargle periodogram analysis. We observed significant rhythmicity for both IBI postural drift and bout rotation, with similar periods close to 27 hours (figure 4F, permutation *p* < 0.001 for both parameters; 26.96 and 27.19 hours periods for IBI drift / bout rotation). We further visualized oscillations in both parameters using Cosinor fits (figure 4G). Notably, their oscillations showed comparable amplitudes (supplementary material, table S4, 2.44 deg versus 2.26 deg), but approximately opposite phases (supplementary material, table S4, 1.04 versus 13.69 hours). These results demonstrate that larvae adjust bout rotation magnitude in coordination with circadian fluctuations in postural drift.

Together, these findings demonstrate that light exerts a masking effect that promotes the exploratory strategy, whereas the circadian clock regulates the magnitude of postural corrections in darkness. Our results disentangle the respective contributions of endogenous rhythms and environmental lighting to the regulation of locomotion in larval zebrafish.

## 4. Discussion

### (a) Circadian and light modulation of balance and navigation

Circadian and lighting cues affect animal behaviour [1–5]. Traditionally, rhythmic activity has been measured with coarse phase markers [12,13,38]. Recent machine-learning video analysis enables detailed kinematic tracking [39,40], but its computational demands limit extended recordings. Conversely, long-term automated classification often lacks fine kinematic resolution [41,42]. Thus, how activity changes translate into behavioural strategies remains largely unknown. We addressed these challenges by combining the simplicity of zebrafish behaviour with high-resolution, real-time pose estimation [20]. This enabled us to define locomotor strategies and identify circadian regulation of postural control.

Classic zebrafish circadian paradigms often use dim light ranging from 3 to 40 lux for the LL condition [12,13,43]. Higher light intensities have been associated with constant activity and loss of circadian rhythmicity [44]. We used the same light intensity in LL as during the LD photophase and observed a dominant effect of light on larval locomotor strategy, consistent with the classic ‘masking effect’ [3,4]. Light-induced masking of behaviour has been reported across species, including mammals [5,45], birds [46], reptiles [47,48], fish [14,18,19,44] and fruit flies [1], but its sensorimotor mechanisms remain unclear. Unlike mammals, zebrafish can detect light directly via the pineal gland and in many other cell types [49,50]. Recent data indicate that both eyes and extra-retinal photosensitive tissues contribute to behavioural modulation [51,52]. Future studies combining loss-of-function approaches with deep phenotyping of light masking could elucidate the functional roles of different light-sensing pathways in locomotor behaviour.

Our results revealed rhythmic fluctuations in postural control kinematics in DD conditions, suggesting that balance behaviour could be modulated by the time of day. This finding adds to the limited evidence of circadian influences on balance [53,54]. Larval zebrafish are an emerging model for studying vestibular function and underlying circuits [21,22,55,56]. Recent work focusing on the zebrafish optomotor response has identified mechanisms through which circadian time modulates circuit dynamics and behaviour [57]. Future studies that integrate kinematic analysis with vestibular circuit measurement will uncover how circadian rhythms shape vestibular processing and balance control.

Together, our work sets the stage for investigating how light and the circadian clock jointly shape motor outputs for postural control in a diurnal vertebrate.

### (b) Compensatory body rotation implies short-term memory of vestibular signals

We found that larvae compensate for postural drift at night with nose-up rotations, enabling longer inactive periods while minimizing unfavourable postures. Unexpectedly, compensatory rotation scaled with passive postural drift rather than inactive-phase duration. This may require larvae to remember either their post-bout posture or postural drift during the inactive phase. Our findings suggest the existence of a short-term memory of vestibular signals on the pitch axis.

Classic heading direction cells integrate angular velocity over time to encode directional and positional signals in the horizontal plane [58–60]. In darkness, vertebrates rely on vestibular input to maintain directional tuning [58]. Unlike horizontal rotation, where only angular velocity can be detected, tilt angles in vertical planes can be inferred from gravitational signals detected by the otolith organs in the inner ear [61]. Although semicircular canals are not yet functional at the stages studied here [62], larval zebrafish otoliths detect gravity and linear acceleration [55]. Our results demonstrate that larvae rely on otolithic signals to restore heading on the pitch axis following prolonged inactivity. Future work may identify neural substrates responsible for the storage of vestibular signals that enable postural compensation.

We imposed a mild balance challenge by ablating lateral-line hair cells, but technical limitations restricted dataset size and prevented us from defining the limits of postural compensation. The mechanism linking lateral-line ablation to reduced swim bladder inflation also remains unclear. Future work could impose stronger balance challenges by covering the water surface with paraffin oil during swim bladder inflation [10] or replacing air above the water with helium to increase nose-up torque.

By characterizing the compensatory body rotation, our work lays the foundation for investigating neural mechanisms of vestibular processing using larval zebrafish.

### (c) Limitations

One statistical limitation of this study is the relatively small number of independent experimental repeats (*n* = 5), which reduces statistical power for repeat-level comparisons. Consequently, some parameters exhibited large effect sizes but only marginal *p*-values. To complement the repeat-level analyses, we examined differences at the level of individual swim bouts, thereby capturing the full distribution of behavioural measurements within each condition. These analyses yielded results that were generally consistent with the repeat-level comparisons and are summarized in the electronic supplementary material.

The behavioural data acquired in this study are subject to several limitations arising from design choices and technical compromises of our apparatus.

First, the size of our behaviour chamber raises questions about the ecological relevance of exploration in this context. Compared with previous studies that assessed exploratory swimming in 35 mm dishes holding 5 ml of water [37,63], our setup contained five to seven larvae swimming in a 30 ml volume of water measuring approximately 50.8 mm (L) × 50.0 mm (H) × 12.7 mm (W) [20]. Given that the volume of a 7 dpf larva, which is approximately 4 mm in length and 0.6 mm in width, can be estimated at around 0.4–0.8 mm^3^ [64], our setup is roughly equivalent to humans diving into an Olympic-sized swimming pool. A larva swimming continuously in the same direction would take 25 to 50 s to cross the chamber. Thus, we reason that we measured kinematic parameters of larvae exploring the environment. Future studies introducing novel environments or points of interest may reveal more complex exploratory strategies.

Second, to achieve high-resolution imaging over days, we recorded larvae that stochastically entered a 20 × 20 mm^2^ field of view in the centre of the arena [20]. This enabled detailed kinematic analysis but limited measurement of global activity, free-running period, rapid responses to light transitions [51] and time spent swimming versus resting on the bottom. Wider-field tracking in deeper arenas would better capture resting, sleep and whole-arena navigation.

Lastly, light intensity varied across apparatuses. Illuminance ranged from 50 to 150 lux and unweighted broadband irradiance spanned 0.61 to 3.91 W m^–2^. We did not detect systematic relationships between irradiance and measurements, but we did not measure each LED strip's spectral output using spectroradiometry. Future work with a full spectral characterization of the light source would facilitate comparison across studies.

## 5. Conclusion

We demonstrated that lighting and circadian cues shape locomotor strategies in larval zebrafish, prioritizing postural control in the dark and environmental exploration in the light. Our findings highlight how the interplay of external and internal cues governs balance and navigation in a freely moving small vertebrate.

## Supplementary Material



## Data Availability

All raw data, analysed data and code for analysis have been uploaded to the Open Science Framework: [25]. All code and data are publicly available under the ‘Dropbox’ tab. Supplementary material is available online [65].
