# Supplementary material for: Lighting and circadian cues shape locomotor strategies for balance and navigation in larval zebrafish
Source: Proc Biol Sci. 2026 Oct 14;293(2080):20261651. doi: 10.1098/rspb.2026.1651 (PMC13678715; doi:10.1098/rspb.2026.1651)
Supplement: Supplementary file 1 [file rspb.2026.1651.zip › rspb.2026.1651/rspb20261651_si_001.pdf]

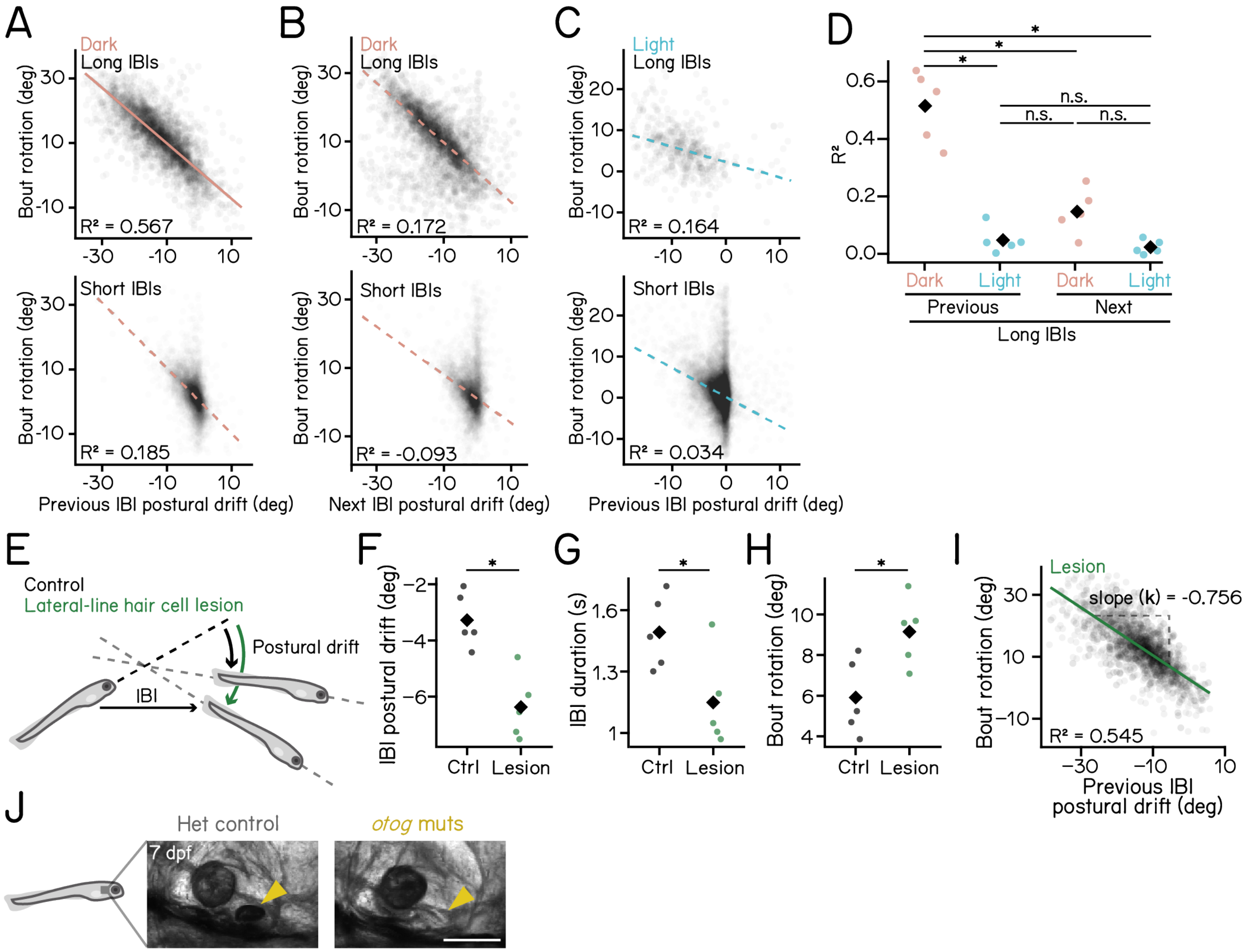

Figure S1: Bout rotations following long swim intervals correlate with IBI postural drifts. Refer to Figure 2.

(A) Scatter plot of bout rotation vs. postural drift during the previous IBI in the dark. Bouts were categorized into long and short IBIs based on the duration of preceding IBIs. Red lines represent the robust bi-square regression fit. Slope of the best-fit line for long IBIs: -0.860, R^2 = 0.567; slope for short IBIs: -0.986, R^2 = 0.185.
(B) Scatter plot of bout rotation vs. postural drift during the following IBI in the dark. Bouts were categorized into long and short IBIs based on the duration of following IBIs. Red lines represent the robust bi-square regression fit. Slope of the best-fit line for long IBIs: -0.874, R^2 = 0.172; slope for short IBIs: -0.712, R^2 = -0.093.
(C) Scatter plot of bout rotation vs. postural drift during the previous IBI in the light. Bouts were categorized into long and short IBIs based on the duration of preceding IBIs. Cyan lines represent the robust bi-square regression fit. Slope of the best-fit line for long IBIs: -0.360, R^2 = 0.164; short IBIs: -0.753, and R^2 = 0.034.
(D) Comparison of R^2 values for regression fits of bout rotation against IBI postural drift (*: adjusted p < 0.001, two-way ANOVA with post-hoc Tukey HSD tests).
(E) Schematics illustrating effects of lateral-line hair cell lesions on IBI postural drifts.
(F–H) Comparisons of bout kinematics between lateral-line lesioned larvae and sham controls: IBI rotation (p = 1.882e-03, Cohen's d = 2.876, t-test) **(F)**, IBI duration (p = 2.992e-02, Cohen's d = 1.667, t-test) **(G)**, and bout rotation (p = 2.028e-02, Cohen's d = 1.826, t-test) **(H)**. Median values for each experimental repeat are plotted as dots. Diamonds indicate group means.
(I) Scatter plot of bout rotation vs. drift during the previous IBI following lesions. Only bouts with long preceding IBI were plotted. The green line represents the robust bi-square regression fit. Slope of the best-fit line for long IBIs: -0.756, coefficient of determination R^2 = 0.545.
(J) *otog* mutants lack the utricular otolith (arrowheads) at 7 dpf. Scale bar: 100 μm.
Middle bouts from 3-bout sequences were selected for IBI postural drift computation. n = 3143/34285 bout during subjective day or the photophase from 105/98 fish over 5 experimental repeats for wild type DD/LD. For (F–H), n = 25008/8982 bouts during subjective day or the photophase from 114/114 fish over 5 experimental repeats for lateral-line hair cell control/lesions. For (I), n = 2994 middle bouts from 3-bout. See also Table 2.

Table 1: Kinematic parameters under DD, LD, and LL during subjective day or the photophase of LD. Refer to Figures 1–2. Data from 5 experimental repeats were pooled to calculate median [IQR]. n = 23875/98722/100674 bouts from 105/98/98 fish for wild type DD/LD/LL. Median test p-values reported for DD day vs. LD day comparisons; Effect size estimated using standardized chi-squared statistics. n = 8336/2994 bouts from 114/114 fish for hair cell control/lesions. n = 3106/1716 bouts from 99/136 fish for *otog* hets/mutants. MAD: median absolute deviation. See Methods for details.

Parameter	Unit	DD day (dark)	LD day (light)	P-value	Effect size	LL day (light)
**Bout parameters**						
Peak speed	mm/s	11.342 [6.238]	13.200 [7.248]	<0.001	0.119	12.711 [6.739]
Displacement	mm	1.440 [0.932]	0.914 [0.711]	<0.001	0.243	0.825 [0.635]
Bout duration	ms	144.578 [54.217]	72.289 [12.048]	<0.001	0.494	66.265 [12.048]
Bout rotation	deg	7.532 [14.454]	1.329 [5.194]	<0.001	0.213	1.717 [5.605]
**Inter-bout interval parameters**						
IBI duration	s	1.584 [2.253]	0.554 [0.392]	<0.001	0.270	0.554 [0.422]
IBI rotation	deg	-4.267 [10.867]	-0.816 [2.038]	<0.001	0.173	-0.752 [2.325]
Bout duration	ms	144.578 [54.217]	72.289 [12.048]	<0.001	0.494	66.265 [12.048]
Bout rotation	deg	7.532 [14.454]	1.329 [5.194]	<0.001	0.213	1.717 [5.605]
**Inter-bout interval parameters**						
IBI duration	s	1.584 [2.253]	0.554 [0.392]	<0.001	0.270	0.554 [0.422]
IBI rotation	deg	-4.267 [10.867]	-0.816 [2.038]	<0.001	0.173	-0.752 [2.325]

Condition	Unit	Median [IQR]	MAD
**Compensation residual (bouts following long IBIs, DD day)**			
Wild type	deg	-0.514 [5.361]	2.660
Hair cell ctrl	deg	-0.253 [5.657]	2.832
Hair cell lesion	deg	-0.250 [6.704]	3.324
otog hets	deg	0.194 [8.164]	4.079
otog mutants	deg	1.651 [15.601]	7.862

Table 2: Correlation of bout rotation and IBI rotation under dark and light. Refer to Figure 2. Wild-type data from 5 experimental repeats were pooled and analysed using bi-square regression. Middle bouts from 3-bout sequences were selected for analysis. n = 3143/34285 bouts during subjective day or the photophase from 105/98 fish for DD/LD over 5 experimental repeats. The hair-cell lesion dataset contains 8336/2994 bouts during subjective day or the photophase from 114/114 fish for control/lesions over 5 experimental repeats.

		**DD day (dark)**			**LD day (light)**		
**IBI length**	**Relation**	Slope	R^2	Bootstrapped p-value	Slope	R^2	Bootstrapped p-value
**Wild type**							
Long	Previous IBI	-0.860	0.567	<0.001	-0.360	0.164	<0.001
	Next IBI	-0.874	0.172	<0.001	-0.648	0.088	<0.001
Short	Previous IBI	-0.986	0.185	<0.001	-0.753	0.034	<0.001
	Next IBI	-0.712	-0.093	<0.001	-0.282	0.011	<0.001
**Lateral-line hair cell lesion control**							
Long	Previous IBI	-0.852	0.451	<0.001			
Short	Previous IBI	-1.131	0.226	<0.001			
**Lateral-line hair cell lesioned**							
Long	Previous IBI	-0.756	0.545	<0.001			
Short	Previous IBI	-1.004	0.371	<0.001			

Table 3: Swim displacement and directional changes of larvae during the subjective day of DD and the photophase of LD. Refer to Figure 3. Data from 5 experimental repeats were pooled to calculate median [IQR]. P-values for DD vs. LD comparisons are from the median test. n = 23875/98722/100674 bouts from 105/98/98 fish for DD/LD/LL for individual bout displacement. n = 7980/120010/132090 sets of 5 consecutive bouts from 105/98/98 fish for DD/LD/LL for average displacement and directional change calculation. Median test p-values reported for DD day vs. LD day comparisons; Effect size estimated using standardized chi-squared statistics. See Methods for details.

Parameter	Unit	DD day (dark)	LD day (light)	P-value	Effect size	LL day (light)
Displacement per second	mm	1.019 [1.117]	1.977 [1.258]	1.067e-154	0.166	1.897 [1.270]
Bout displacement	mm	1.440 [0.932]	0.914 [0.711]	<0.001	0.243	0.825 [0.635]
Bout direction	deg	12.971 [30.907]	-0.095 [19.875]	<0.001	0.183	0.828 [20.144]
Directional change per second	deg	1.959 [2.391]	4.005 [6.197]	1.318e-129	0.151	3.800 [5.775]
Directional change between bouts	deg	4.809 [8.064]	3.792 [7.043]	4.199e-49	0.041	3.648 [6.552]

Table 4: Circadian rhythm parameters for 2-bout latter bout duration, IBI rotation, and bout rotation. Refer to Figure 4. Lomb–Scargle and cosinor analyses were performed on detrended 1 h per-fish median values. Lomb–Scargle peaks were searched between 12 to 36 h periods, and empirical peak p-values were estimated with 1000 permutations. Cosinor fits used each condition's dominant Lomb–Scargle period. Phase is the Cosinor acrophase in hours relative to the aligned experiment start, modulo the fitted period.

Parameter	Unit	Cond.	Fish n	Lomb–Scargle			Cosinor			
				Period (h)	Power	Perm. P	Amplitude	Phase (h)	R^2	P-value
Bout duration	ms	DD	12	27.190	0.181	<0.001	17.345	15.690	0.184	<0.001
		LD	16	24.135	0.550	<0.001	—	—	—	—
		LL	12	24.690	0.033	<0.001	2.542	0.147	0.033	<0.001
IBI postural drift	deg	DD	12	26.962	0.072	<0.001	2.437	1.035	0.074	<0.001
		LD	16	23.779	0.398	<0.001	—	—	—	—
		LL	12	25.271	0.088	<0.001	2.352	10.986	0.089	<0.001
Bout rotation	deg	DD	12	27.190	0.061	<0.001	2.260	13.686	0.061	<0.001
		LD	16	23.779	0.371	<0.001	—	—	—	—
		LL	12	25.074	0.078	<0.001	2.161	23.972	0.078	<0.001
